# Evaluating the impact of an intensive education workshop on evidence-informed decision making knowledge, skills, and behaviours: a mixed methods study

**DOI:** 10.1186/1472-6920-14-13

**Published:** 2014-01-17

**Authors:** Jennifer Yost, Donna Ciliska, Maureen Dobbins

**Affiliations:** 1 School of Nursing, McMaster University, Hamilton, Ontario, Canada

**Keywords:** Knowledge translation, Educational workshop, Evidence informed decision making, Evidence based practice, Knowledge, Skills, Behaviours, Health professionals

## Abstract

**Background:**

Health professionals require a unique set of knowledge and skills in order to meet increasing expectations to use research evidence to inform practice and policy decisions. They need to be able to find, access, interpret, and apply the best available research evidence, along with information about patient preferences, clinical expertise, and the clinical context and resources, to such decisions. This study determined preferences for continuing education following an intensive educational workshop and evaluated the impact of the workshop on evidence informed decision making (EIDM) knowledge, skills, and behaviours.

**Methods:**

An explanatory mixed methods, longitudinal study design was implemented among a convenience sample of various health care professionals attending the workshop. EIDM knowledge, skills, and behaviours were quantitatively measured at baseline and six month follow-up, with EIDM knowledge and skills measured additionally immediately following the educational workshop (post-test measurement). To determine participants preferences for continuing education, data were collected using quantitative survey (post-test measurement) and qualitative (individual telephone interviews after six-month follow-up) methods.

**Results:**

EIDM knowledge and skills increased significantly from baseline to immediately following the intervention [5.6, 95% CI (3.7, 7.4), *P* < 0.001] and from baseline to six-month follow-up [3.7, 95% CI (2.1, 5.3), *P* < 0.001], with a significant decrease from immediately following the intervention to six-month follow-up [-1.9, 95% CI (-3.5, -0.3), *P* 0.018]. EIDM behaviours increased, but not significantly, from baseline to six-month follow-up [1.7, 95% CI (-0.3, 3.8), *P* 0.095]. At baseline and six-month follow-up there was a weak, non-significant positive correlation between EIDM knowledge and skills and EIDM behaviours (*r* = 0.29, *P* 0.069 and *r* = 0.24, *P* 0.136, respectively). Over time there was a shift in preferences for timing and frequency of online continuing education strategies. Willingness to participate in continuing education, however, remained evident.

**Conclusions:**

An intensive educational workshop shows promise for increasing EIDM knowledge and skills. Increasing EIDM knowledge and skills may promote the capacity of health professionals to use research evidence when making practice and policy decisions and, in turn, lead to positive patient outcomes.

## Background

Health professionals are increasingly expected to use research evidence in practice, program development, and policy decisions to improve client and system outcomes. This involves finding, accessing, and interpreting the best available research evidence and then adapting, implementing, and evaluating its impact. Simply put, the expectation is to put knowledge into action [1]. In order to put knowledge into action, the knowledge and skills associated with evidence-informed decision making (EIDM) are necessary. Such knowledge and skills include: asking the question, searching for the best available evidence, assessing the quality of the evidence through critical appraisal, interpreting the evidence, determining the relevance of the evidence to practice, program, and policy decisions, and acting on the evidence if and when appropriate [2].

EIDM knowledge and skills have been shown to be limited among health care professionals [3,4]. Several systematic reviews have examined the effectiveness of interventions to increase EIDM. Findings have suggested that active interventions result in small to moderate improvements in EIDM behaviours and patient outcomes. Such active interventions include reminders, educational outreach, opinion leaders, audit and feedback, as well as point-of-care computer reminders [5-19].

For the past several years, the Canadian Centre for Evidence-Based Nursing (http://ccebn.mcmaster.ca/), in the School of Nursing at McMaster University, has been offering a week long intensive educational workshop aimed at increasing participants’ knowledge, skill, and confidence to engage in EIDM. Although active strategies, such as this EIDM Workshop, are perceived as effective mechanisms for improving knowledge, limited evaluation of their impact on EIDM knowledge, skill, and behaviour has occurred [20]. If successful, such improvements in EIDM knowledge, skills, and behaviors have the potential to lead to the adoption of the most effective and cost-efficient interventions to improve the health outcomes of individuals and communities [21,22]. Therefore, the purpose of the study was to address the following research questions among health professionals involved in decision-making: 1) Does an intensive educational workshop increase EIDM knowledge and skills? 2) Does an intensive educational workshop increase EIDM behaviours? 3) Is there a relationship between EIDM knowledge and skills and EIDM behaviours before and after an intensive educational workshop? and 4) What are participants’ preferences for continuing education following an intensive educational workshop to sustain EIDM knowledge, skills, and behaviours?

## Methods

This study was approved by the by Hamilton Health Sciences/Faculty of Health Sciences Research Ethics Board.

### Design and sample

An explanatory mixed methods, longitudinal study design was used to evaluate the impact of an intensive educational workshop upon individuals’ ability to engage in EIDM [23]. Quantitative methodology was used to determine if the intensive educational workshop increased EIDM knowledge, skills, and behaviours. Qualitative methodology was used to determine participants preferences for continuing education following an intensive educational workshop to sustain EIDM knowledge, skills, and behaviours. These methodologies, including data collection, outcome measurement, and data analysis, will be described in further detail.

A convenience sample of attendees (n = 51) at the 2010 EIDM Workshop were invited to participate. Participants were recruited through information posted on CCEBN’s website, broadly distributed email announcements, and personal contacts. Participation was not restricted and was open to individuals of all skills levels and professions.

### EIDM workshop

The EIDM Workshop, a five-day intensive educational workshop, was held in May 2010. Learning methods included large and small group (8 to 15 participants) sessions, individual study time, and opportunities to work with a trained librarian. Participants attending the workshop receive reading materials in advance of the workshop. These materials included background reading and studies used to practice critical appraisal techniques. The materials were altered slightly to be discipline specific. For example, participants in the nursing group received slightly different materials than those in the groups that included participants from public health, library services, and nursing education. Large and small group sessions were delivered by McMaster University faculty with expertise in EIDM. Participants spent a total of 4 hours in large group sessions, 18 hours in small groups sessions, and were provided with extended breaks to allow for individual study time. While large group sessions used didactic and interactive teaching/learning strategies to cover broad content related to EIDM, small group sessions used active teaching/learning strategies to focus on searching for and accessing evidence and critical appraisal of the evidence. For example, participants might work in small groups of two or three to compare responses to critical appraisal criteria before sharing with the larger small group. Each small group conducted critical appraisal of therapy/intervention studies, systematic reviews/meta-analyses, and practice guidelines. Additional file 1 provides an overview of the topic areas, objects, and resources used in the large and small group sessions. As a result of the workshop, it is expected that participants will gain knowledge and skills in searching for, accessing, and critically appraising the relevance and quality of evidence in order to interpret, apply the evidence to decision making, and identify strategies to implement evidence-informed decisions in the local context.

### Outcome measures

EIDM knowledge is conceptualized as learner’s retention of facts and concepts about the steps of EIDM, while EIDM skills represent the application of this knowledge [24,25]. A recent systematic review by Shaneyfelt and colleagues [24] identified multiple tools to evaluate the knowledge and skills associated with EIDM. The majority of these instruments, however, evaluate only the knowledge and skills for searching and critical appraisal in physician populations [25]. Among tools with acceptable validity and reliability [25], the Fresno Test/Adapted Fresno Test [26,27] and Berlin Questionnaire [28] assess the first four steps of EIDM (ask, search, appraise, and integrate). These assessments have focused on searching and appraisal of primary research evidence among medical students and occupational therapists [24,25,29].

To address this gap, the researchers developed a questionnaire (EIDM Skills Tool) to measure EIDM knowledge and skills. The EIDM Skills Tool was designed around scenarios with broad applicability and inclusive of all of the EIDM knowledge and skills. Within the Classification Rubric for Evidence Based Practice (EBP) Assessment Tools in Education (CREATE), the EIDM Skills Tool objectively assesses knowledge in all EIDM steps (asking the question, searching for the best available evidence, assessing the evidence for quality through critical appraisal, interpreting the evidence, and determining the relevance of the evidence to decisions) and skill in asking the question, assessing the evidence for quality through critical appraisal, and interpreting the evidence. [24]. Content validity was established with experts in the field for this questionnaire which consists of 18 open-ended and multiple-choice format questions. Content of the questions includes the following: 1) one question on formulating questions using the PICO format [P: patient(s) or population(s); I: intervention; C: comparison; O: outcome(s)]; 2) one question on search strategies associated with the 6S Pyramid [30]; 3) nine questions on the critical appraisal of therapy/interventions studies, including concealment, blinding, intention-to-treat analysis, effect size, and application of results; 4) seven questions on the critical appraisal of systematic reviews/meta-analyses, including search strategy, quality assessment criteria, effect size, and application of results. Scoring for the EIDM Skills Tool was done with a marking key and consisted of summing responses to the items for a total score that ranged from 0 to 36.

The EBP Implementation Scale and EBP Implementation in Public Health Scale were used to evaluate the degree to which participants engaged in behaviours associated with EIDM. Within the CREATE framework, both EBP Implementation Scales subjectively assesses behaviours associated with all the steps of EIDM [24]. Permission to use these scales was obtained from Melynk and Fineout-Overholt [31]. Both EBP Implementation Scales ask participants to respond to 18 items on a five-point scale (0 times, 1–3 times, 4–5 times, 6–7 times, ≥ 8 times) by indicating how often in the past eight weeks they performed a range of EIDM behaviours. On both of the scales, the 18-items ask the same question in the same order. The EBP Implementation in Public Health Scale, however, uses terminology consistent with public health. For example, the term such as “health care providers”, “patients”, and “clinical practice”, are reflected respectively as “public health professionals”, “clients”, and “public health practice” on the EBP Implementation in Public Health Scale. Scoring for both EBP Implementation Scales consists of summing responses for the 18 items for a total score than can range from 0 to 72 [31].

The EBP Implementation Scale has demonstrated acceptable content and construct validity, as well as high internal consistency reliability and intra-scale correlation (Cronbach’s α 0.96 and Spearman Brown *r* 0.95, respectively) [31]. The researchers did conduct a pilot study to establish reliability for the EBP Implementation in Public Health scale. Due to a small sample size of target users in the pilot study, reliability measures were unable to be determined.

A secondary aim of this study was to determine participants’ preferences for continuing education following an intensive education workshop to sustain EIDM knowledge, skills, and behaviours. Data on preferences for continuing education were collected using a quantitative questionnaire and individual semi-structured interviews. The quantitative questionnaire asked participants about their willingness to participate in continuing education and in different formats (i.e. face-to-face workshops, various online strategies). These questions were rated on a seven-point Likert scale ranging from ‘strongly disagree’ to ‘strongly agree’. Additional questions asked about preferences for the timing of continuing education. Interviews were conducted using the interview guide (Additional file 2).

### Data collection

Prior to attending the workshop, all EIDM Workshop registrants received a copy of the Information and Consent Form along with the workshop materials. On the first day of the EIDM Workshop, prior to any learning activities, signed Information and Consent forms were obtained from those agreeing to participate in the study. Participants subsequently completed a standard demographic form, along with the EIDM Skills Tool and EBP Implementation Scale or EBP Implementation in Public Health Scale. EIDM Workshop registrants who indicated that they worked in public health when registering for the workshop completed the EBP Implementation in Public Health Scale, all others completed the EBP Implementation Scale. This was considered the baseline measurement (May 2010).

On the last day of the EIDM Workshop (five days later), post-test measurement was conducted. Participants completed the EIDM Skills Tool and the quantitative questionnaire about continuing education preferences.

Six-months following the baseline measurement (November, 2010), participants completed the EIDM Skills Tool and EBP Implementation Scales. Whereas previous data collection was done via paper-and-pencil, participants completed the six-month follow-up online via a secure website. Telephone interviews were then conducted by one of the researchers (JY) following the six-month data collection using a standardized interview guide developed specifically for this study. Interviews occurred at a time that was agreed upon in advance of the interview and lasted approximately 30 minutes.

### Data analysis

Quantitative data was analysed using IBM SPSS Statistics 20. Descriptive statistics were performed on demographic data, EIDM knowledge and skills scores (collected via EIDM Skills Tool), EIDM behaviours (collected via EBP Implementation scales), and data on preferences for continuing education collected via quantitative questionnaire. To determine if there was a change in EIDM knowledge and skills from baseline to six-month follow up, repeated measures analysis of variance (ANOVA) with post-hoc tests using Bonferroni correction was performed. The analysis included each participant’s total score on the EIDM Skills Tool as the dependent variable and determined differences across the three time points (baseline, post-test, six-month follow up). The proportion of EIDM knowledge and skills retained over time was also calculated using the following formula: (six-month follow-up measurement – baseline measurement) divided by (post-test measurement – baseline measurement). Paired *t* tests were performed to determine if there was a change in EIDM behaviours. To determine if there was a relationship between EIDM knowledge and skills and EIDM behaviours, Pearsons correlation was performed.

The proportion of EIDM knowledge and skills retained over time was analysed using data for only participants who completed the EIDM Skills Tool at all three time points (baseline, post-test, and six-month follow-up measurement). Retention scores of three participants representing outliers (> ± 100%) were further excluded from the analysis. Missing data was then imputed for all other analyses. For missing data at baseline the average score at baseline was imputed and for missing data at post-test measurement and six-month follow-up, the last observation was carried forward. All analyses used the total score on the EIDM Skills Tool and/or EBP Implementation scales.

Interviews were audio-taped and transcribed verbatim. NVivo software was used to explore and code the qualitative data which was then grouped into themes. Quantitative and qualitative results were merged during interpretation. Themes identified during qualitative data analysis were compared with quantitative results to explain findings about continuing education preferences.

## Results

### Sample characteristics

**Figure 1:**
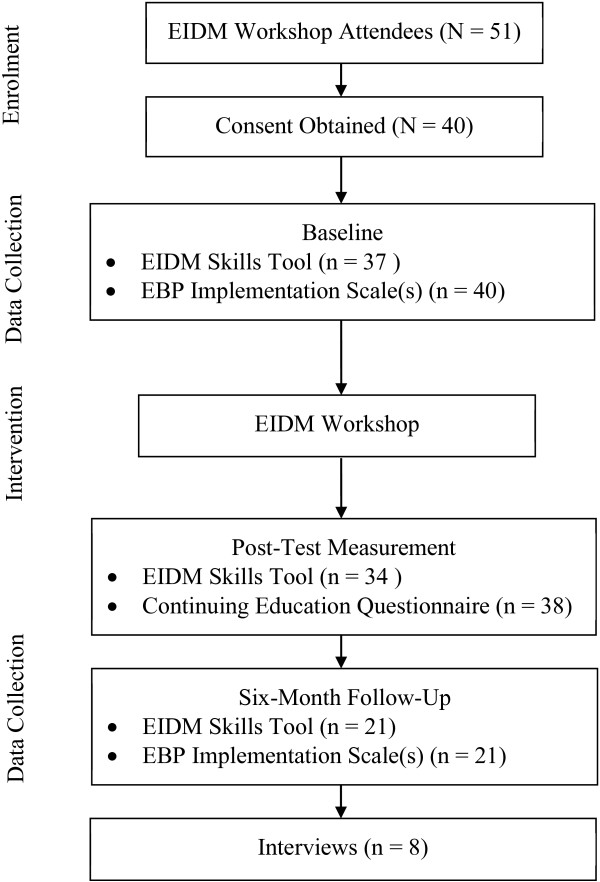
**Flow of participants.** This file provides the flow of participants through the study.

Of the 51 EIDM Workshop attendees, 40 attendees consented to participate in this study (Figure 1). Table 1 provides an overview of the demographic information for the participants, while Table 2 provides the response rate of the participants at each time point in the study for quantitative data collection. The majority of participants were female, from Ontario, Canada, and worked in public health. There was however, representation from other provinces which is historically typical of the EIDM workshop. In addition to participants in public health, participants also included faculty from McMaster University or its partner sites (Conestoga College and Mohawk College), nurses, advanced practice nurses, and librarians.

**Table 1:** Demographic and other characteristics

**Demographic**		
Age in years (mean, SD)	44.3	9.2
Women (n,%)	40	100
Province of residence (n,%)		
Ontario	33	82.5
Manitoba	2	5.0
New Brunswick	2	5.0
Nova Scotia	2	5.0
Saskatchewan	1	2.5
Highest educational degree (n,%)		
Diploma	0	0.0
Baccalaureate	20	50.0
Masters	18	45.0
Other	1	2.5
Missing	1	2.5
Professional designation (n,%)		
RN	25	62.5
APN	1	2.5
MD	1	2.5
Librarian	2	5.0
Other	10	25.0
Missing	1	2.5
Employment setting (n,%)		
Academic	9	22.5
Acute Care	2	12.5
Long Term Care	0	0.0
Public Health/Community	23	57.5
Other	2	5.0
Missing	1	2.5
Main job function (n,%)		
Executive officer	2	5.0
Associate medical officer of health	1	2.5
Program manager/administrator	14	35.0
Direct service/care provider	5	12.5
Research	2	5.0
Policy development/analysis	1	2.5
Faculty	8	20.0
Other	6	15.0
Missing	1	2.5
Previous experience (n,%)		
Yes	39	97.5
No	1	2.5
Previous experience* (n,%)		
Taken a session	9	22.5
Read a book	8	20.0
Taken a workshop	5	12.5
Taken an undergraduate course	12	30.0
Taken a graduate course	9	22.5
Participated in a journal club	3	7.5
Taught as a tutor	5	12.5

*Participants were able to select more than one response.

**Table 2:** Response rates at baseline, post-test, and six-month follow-up

	**Baseline n (%)**	**Post-test n (%)**	**Six-month follow-up n (%)**
**EIDM Knowledge/ Skills**	37 (92.5%)	34 (85.0%)	21 (52.5%)
**EIDM Behaviours**	40 (100%)	n/a	21 (52.5%)

Just over half of the participants reported having a baccalaureate degree as their highest degree, with approximately 46% of participants having master’s degree. An overwhelming majority of the participants reported having previous experience with EIDM, however the degree of experience varied. Among participants, 30% reported taking an undergraduate course and 22.5% reported taking a graduate course that included EIDM, 12.5% reported taking a similar workshop about EIDM, and 7.5% reported participating in a journal club.

### Change in EIDM knowledge and skills

There was a significant increase in EIDM knowledge and skills from baseline to post-test measurement and baseline to six-month follow-up. On average total EIDM knowledge and skills score increased by 5.6 points [95% CI (3.7 to 7.4), *P* < 0.001] from baseline to post-test measurement and by 3.7 points from baseline to six-month follow-up [95% CI (2.1 to 5.3), *P* < 0.001] (Tables 3 and 4). From post-test measurement to six-month follow-up there was, however, a significant decrease in EIDM knowledge and skills [-1.9, 95% CI (-3.5 to -0.3), *P* 0.018] (Tables 3 and 4). Additionally, the proportion of EIDM knowledge and skills retained over time for participants (n = 15) was 43.7% (range = -46.5% to 97.7%).

**Table 3:** Total scores at baseline, post-test, and six-month follow-up [mean (SD)] (N = 40)

	**Baseline**	**Post-test**	**Six-month follow-up**
**EIDM Knowledge/Skills**	9.5 (3.4)	15.1 (5.2)	13.2 (5.1)
**EIDM Behaviours**	14.5 (11.9)		16.2 (11.3)

**Table 4:** Mean differences in total scores over time (N = 40)

	**Mean difference**	**95% CI**	***P***
*Baseline- to Post-Test*			
EIDM Knowledge/Skills	5.6	(3.7 to 7.4)	< 0.001
*Post-Test to Six-Month Follow-up*			
EIDM Knowledge/Skills	-1.9	(-3.5 to -0.3)	0.018
*Baseline to Six-Month Follow-up*			
EIDM Knowledge/Skills	3.7	(2.1 to 5.3)	< 0.001
EIDM Behaviours	1.7	(-3.8 to 0.3)	0.095

CI = Confidence Interval.

### Change in EIDM behaviours

There was a non-significant increase in EIDM behaviours from baseline to six-month follow-up. Scores on the EBP Implementation scales increased from 14.48 at baseline to 16.0 at post-test measurement [1.7, 95% CI (-0.3 to 3.8), *P* 0.095] (Tables 3 and 4).

### Relationship between EIDM knowledge and skills and EIDM behaviours

Results showed a non-significant, weak positive correlation between EIDM knowledge and skills and EIDM behaviours at baseline (*r* = 0.29, *P* 0.069) and six-month follow-up (*r* = 0.24, *P* 0.136).

### Preferences for continuing education

At the end of the EIDM workshop, 97% of the participants completing the quantitative survey about preferences for continuing education indicated that they would be interested in participating in continuing education following the workshop. Among those who participated in the interviews conducted after the six-month follow-up quantitative data collection (n = 8), this willingness to participate in continuing education remained a theme among the majority of participants. Only one interviewee expressed that he/she, personally, would not benefit from continuing education.

In terms of content, there was some disconnect between areas that participants indicated they wanted to better understand at the end of the workshop versus six-months after attending the workshop. At the end of the workshop, participants wanted more review of all of the topics covered in the workshop; but at six months they wanted new content related to critical appraisal of other types of studies.

At the end of workshop and after six-months following the workshop, participants preferred one to two day workshops. At six months, respondents preferred that these workshops occur at their organization. In terms of online continuing education, strategies such as webcasts, online learning modules, and discussion boards were identified at both time points. Although spending one to two hours at a time for online strategies was identified at both time points, six-months following the workshop there was a much broader range in participants’ preferences. Preferences varied depending on the time expected for preparation and participation, as well as and the method of delivery of the content (i.e. didactic versus interactive). Half of the participants completing the quantitative survey at the end of the workshop indicated that they would participate about once per month; however six-months afterwards participants were reporting longer intervals of six weeks to three months.

Interviews also explored perceived barriers and facilitators to participating in continuing education. Reported barriers and facilitators varied by the delivery method, but common themes included time, geography, and relevance. Within the theme of time, participants indicated that their participation in sessions would be influenced by length of time for the sessions and the ability to commit to the time for the session due to workload and other commitments. In terms of geography, participants expressed that the location of face-to-face sessions or the time zone differences for online learning should be considered. Lastly, participants identified that the sessions would need to be relevant and support continued development of knowledge and skills required in functions associated with their employment, in order to participate.

## Discussion

An increase in the EIDM knowledge and skills following an educational workshop in this study is consistent with findings of previous systematic reviews [16,32-34]. These reviews included educational workshops delivered as stand-alone workshops or as part of a multifaceted intervention in populations largely representative of medical students, interns, or residents. Although these reviews suggest that there is substantial evidence that knowledge improves following educational workshops, there is a lack of information about similar improvements in skills [32]. It remains challenging to apply the findings of this study to previous studies given the range of interventions to promote EIDM knowledge and skills and the numerous tools to measure EIDM knowledge and skills [25].

This study considered an intensive educational workshop delivered to a broad range of professionals working within health care, including nurses, public health professionals, and librarians, and other studies with similar populations and interventions support the finding that educational workshops promote EIDM knowledge [35-38]. Using similar designs Shuval and colleagues [37] and Awonuga and colleagues [35] found significant improvements in knowledge immediately after their workshops which were approximately one day in duration among primary care physicians and varied health professionals, respectively. A randomized control trial by Forsetlund and colleagues [36] revealed that public health physicians who attended a one to five day workshop also had significant change in knowledge about sources of information and EIDM concepts at the end of the workshop than participants who did not attend the workshops. In addition to evaluating knowledge, Taylor and colleagues [38] also evaluated critical appraisal skills relevant to systematic reviews following a half-day workshop among a varied group of health professionals. Although the intervention resulted in significant increase in knowledge, only skills in appraising the results of systematic reviews - and not the methodology or relevance/generalizability – improved significantly.

A positive retention of EIDM knowledge and skills over time was also demonstrated in this study. Despite a lack of studies conducted in comparable populations with a similar content focus, interventions, and outcomes, general educational research supports the finding in this study that approximately half of the EIDM knowledge and skills originally learned was remembered by participants. A higher percentage relative to studies in general education, the percentage of knowledge retained in this study may be explained by active learning strategies used in the workshop, the significant increase in learning during the workshop reflected in the change from baseline to post-test measurement, and a relatively short retention interval of 6 months. What is not known, is the degree to which learning that occurred following the workshop may have also positively influenced the EIDM knowledge and skill retention of participants [39].

Despite a significant increase in EIDM knowledge and skills and a positive retention of EIDM knowledge and skills over time, this study did not demonstrate similar improvement in EIDM behaviours following an education workshop. Although this finding is consistent with those of the primary studies by Forsetlund and colleagues [35] and Taylor and colleagues [37], it is not consistent with those of recent systematic reviews which suggest that educational workshops result in small to moderate improvements in EIDM behaviours [8,9,33,40]. Similar to the evidence for changes in EIDM knowledge and skills, these recent systematic reviews also are largely representative of physician populations (including medical students, interns, and residents) and include comparisons of educational workshop interventions that may have been components of multi-faceted interventions.

As an intervention, the intensive educational workshop delivered in this study was aimed primarily at overcoming a barrier to engaging in EIDM behaviours, lack of EIDM knowledge and skills. There are possible explanations for a lack of significant change in EIDM behaviours. First, given the small sample size, insufficient power could have precluded finding a significant change in EIDM behaviours. In addition, it is also possible that participants over-reported engaging in EIDM behaviours at baseline. Although over-reporting may be a limitation of self-reported measures, in this study it is likely that through the week-long workshop participants gained insight as to what is entailed in the EIDM behaviours they were being asked about. For example, one of the questions on the EBP Implementation Scales asks participants how many times they have ‘critically appraised evidence from a research study.’ At baseline participants may have perceived that they were engaged in ‘critically appraising’ research evidence, but as a result of the week-long workshop they gained a better understanding of the criteria for critical appraisal of various types of research evidence and how to apply this criteria. Similar observations of variations in perceptions have been reported by knowledge brokers working with similar types of decision makers as the participants in this study [41]. We also hypothesized that there would be a positive relationship between EIDM knowledge and skills and EIDM behaviours – the more knowledge and skills professionals have the more frequently they will engage in EIDM behaviours. Although a positive relationship was demonstrated, it was not statistically significant and the possible over-reporting of EIDM behaviours may also explain this finding.

The willingness to participate in continuing education was evident immediately following the week-long workshop and remained evident after six-months following the workshop. Preferences for the time and frequency of online continuing education strategies appeared to shift during this timeframe. These findings are helpful for designing and implementing interventions to promote EIDM knowledge, skills, and behaviours.

Given the limitations, the generalizability of the findings of this study should be interpreted with a degree of caution. First, participants represent a small convenience sample. Furthermore, qualitative interviews were conducted with participants who agreed to be interviewed which precluded the ability for data collection to continue until saturation occurred. Participants were also motivated and supported by their institution as they were attending a week-long intensive educational workshop that was associated with a cost (either paid by themselves or their employer) and time away from work (for the most part, paid leave). Originally the researchers had intended to use possible attendees who were wait-listed for the workshop as a control group, but all attendees who expressed interest in the workshop were enrolled in the workshop. Although the absence of a control group could affect the change at post-test and six-month follow-up, it is much more of a serious threat to the assessment at the six-month follow-up. Without a control group, therefore, it is possible that the significant increase in knowledge and skills from baseline to six-month follow-up could have been due to factors other than the EIDM workshop, such as receiving additional support following the EIDM workshop to promote EIDM knowledge and skills. For example, participants included faculty responsible for teaching students EIDM knowledge and skills and practitioners working at a local public health unit that provided and EIDM journal club for those attending the EIDM workshop. In addition, EIDM behaviours were measured using a tool with documented validity and reliability [31], but were self-reported and not objectively measured.

## Conclusions

This study contributes to the evidence that short (one week) educational workshops promote the retention of EIDM knowledge and skills over time among a broad range of health professionals. The findings demonstrating a non-significant change in EIDM behaviours, however, are difficult to interpret. Future research is needed to determine if educational workshops, delivered as stand-alone workshops or as part of multifaceted interventions that provide continuing education, are effective for sustaining EIDM knowledge and skills and promoting EIDM behaviours. When designing and implementing future interventions to promote EIDM knowledge, skills, and behaviours, barriers and facilitators for continuing education interventions should be considered. Sustaining EIDM knowledge and skills may, in turn, promote the capacity of health professionals to engage in EIDM behaviours. Using research evidence when making practice and policy decisions can, in turn, lead to positive patient outcomes.

## Abbreviations

ANOVA: Analysis of variance; CCEBN: Centre for Evidence Based Nursing; EBP: Evidence based practice; EIDM: Evidence-informed decision making; PICO: Population, intervention, comparison, outcome.

## Competing interests

The authors declare that they have no competing interests.

## Authors' contributions

JY contributed to the design of the study, was responsible for the implementation of the study and data analysis, contributed to interpretation of the data, as well as drafted and critically revised the manuscript. DC and MD contributed to the design of the study, study implementation, interpretation of the data, and revisions of the manuscript. All authors read and approved the final manuscript.

## Pre-publication history

The pre-publication history for this paper can be accessed here:

http://www.biomedcentral.com/1472-6920/14/13/prepub

## Supplementary Material

Additional file 1 EIDM Workshop Session Topic Areas, Aims, and Resources This file provides an overview of the topics, aims, and resources used to deliver the EIDM Workshop intervention. Click here for file

Additional file 2 **Interview guide.** This file provides the Interview Guide that was used to conduct interviews with participants. Click here for file
