# Supplementary material for: Evaluating the impact of an intensive education workshop on evidence-informed decision making knowledge, skills, and behaviours: a mixed methods study
Source: BMC Med Educ. 2014 Jan 17;14:13. doi: 10.1186/1472-6920-14-13 (PMC3929552; doi:10.1186/1472-6920-14-13)
Supplement: Additional file 1 — EIDM Workshop Session Topic Areas, Aims, and Resources This file provides an overview of the topics, aims, and resources used to deliver the EIDM Workshop intervention. [file 1472-6920-14-13-S1.pdf]

Additional File 1: EIDM Workshop Session Topic Areas, Aims, and Resources

<table>
<tr><th rowspan="2">Topic Area</th><th rowspan="2">Aims of Large Group Sessions</th><th colspan="2">Aims of Small Group Sessions</th><th rowspan="2">Resources</th></tr>
<tr><th>All Groups</th><th>Dependent per Group</th></tr>
<tr><td>Introduction to EIDM</td><td>• The historical perspective is described
• The components of the Model for EIDM [1; 2] are described
• The steps of EIDM [2] are described
• “What is evidence?” is discussed
• Factors influencing EIDM are identified and discussed</td><td>• Define evidence-informed decision making (EIDM).
• Describe the potential impact of EIDM in practice, program, and policy decisions.
• Describe the current limitations of EIDM.
• Identify facilitators and barriers to EIDM at individual, organizational, and policy levels.</td><td></td><td></td></tr>
<tr><td>Searching for the Best Evidence</td><td>• Format for framing questions is discussed
• The Hierarchy of Pre-Appraised Evidence [3] is presented
• The use of pre-appraised sources of evidence is explained and demonstrated</td><td>• Develop answerable quantitative questions using the PICO [P = patient(s)/population; I = intervention; C = comparison, O = outcome(s)] and qualitative questions PS [P = patient(s)/population; S = situation] acronyms
• Identify databases and sources for locating the best available evidence, including pre-</td><td></td><td></td></tr>
</table>

	• Resources to search for the best evidence are explained an demonstrated	processed evidence<br>• Describe and implement search strategies for accessing evidence through major databases and sources.<br>• State relevant questions using the components of PICO and PS		
Critical Appraisal	•	• Identify the features, advantages, and disadvantages of different quantitative study designs<br>• Differentiate between a systematic review, a meta-analysis, and a meta-synthesis<br>• Identify sources of bias in quantitative research study designs<br>• Understand and apply the critical appraisal criteria relevant to quality assessment of: a) therapy/intervention studies, b) systematic reviews/meta-analysis, and c) practice guidelines<br>• Understand and interpret important statistical concepts such as odds ratios, relative risks, numbers needed to treat, and confidence intervals.<br>• Interpret the meaning and precision of study results, including the size and precision	• Identify different types of qualitative research study designs<br>• Distinguish between association and causation.<br>• Distinguish between clinical and health services interventions.<br>• Understand and apply the critical appraisal criteria relevant to quality assessment of a) qualitative studies, b) causation/harm studies, and c) health services intervention studies	Online Learning Modules<br>• National Collaborating Centre for Methods and Tools (http://learning.nccmt.ca/en/)<br>• Canadian Institute of Health Research (http://www.cihr-irsc.gc.ca/e/39128.html)<br><br>Critical Appraisal Tools<br>• Compendium of Critical Appraisal Tools for Public Health Practice [4]

		of the treatment effect and understanding of figures produced for meta-analyses<br>• Discuss the process of adopting or adapting practice guidelines		
Knowledge Transfer & Dissemination	• Ways in which research evidence is used are identified<br>• Resources for EIDM to address organizational and individual barriers are identified	• Apply study results to decisions about clinical practice questions<br>• Compare and contrast models for knowledge transfer and dissemination used by health care practitioners and policy makers.<br>• Identify effective implementation strategies to facilitate EIDM in for decisions in education, patient care, management, and health care/policy decisions<br>• Explore and critique advanced strategies to facilitate EIDM, such as practice guidelines and decision support tools		• Applicability and Transferability of Evidence Tool [5]<br>• From Research to Practice: A Knowledge Transfer Planning Guide [6]<br>• Registered Nurses Association Toolkit: Implementation of Clinical Practice Guidelines [7]

References

1. DiCenso A, Ciliska D, Guyatt G: **Introduction to evidence-based nursing**. In *Evidence-based nursing: A guide to clinical practice*. Edited by DiCenso A, Ciliska D, Guyatt G. S. Louis, MO: Elsevier Mosby; 2005: 3-1).

2. National Collaborating Centre for Methods and Tools. **A Model for Evidence-Informed Decision-Making in Public Health**. 2012 http://www.nccmt.ca/pubs/FactSheet_EIDM_EN_WEB.pdf

3. DiCenso A, Bayley :L, Haynes B: Accessing pre-appraised evidence: **Fine-tuning the 5S model into a 6S model**. *Evidence-Based Nursing* 2009, ***12***:99-101.

4. Ciliska D, Thomas H, Buffet C: **A Compendium of Critical Appraisal Tools for Public Health Practice (Revised)**. 2012 http://www.nccmt.ca/pubs/CompendiumToolENG.pdf

5. Buffett C, Ciliska D, Thomas H: **Can I use this evidence in my program decision? Assessing applicability and transferability of evidence**. 2007 http://www.nccmt.ca/publications/9/view-eng.html

6. Reardon R, Lavis J, Gibson J:**From Research to Practice: A Knowledge Transfer Planning Guide**. 2006 http://www.iwh.on.ca/system/files/at-work/kte_planning_guide_2006b.pdf

7. Registered Nurses Association of Ontario: **Toolkit: Implementation of clinical practice guidelines**. 2002 http://www.rnao.org/Storage/12/668_BPG_Toolkit.pdf
