# Supplementary material for: Evaluating the impact of an intensive education workshop on evidence-informed decision making knowledge, skills, and behaviours: a mixed methods study
Source: BMC Med Educ. 2014 Jan 17;14:13. doi: 10.1186/1472-6920-14-13 (PMC3929552; doi:10.1186/1472-6920-14-13)
Supplement: Additional file 2 — Interview guide. This file provides the Interview Guide that was used to conduct interviews with participants. [file 1472-6920-14-13-S2.pdf]

Additional File 2: Interview Guide

Date of Interview: ________________ *(please note in day/month/year format)*
Telephone Number: _______________ *(please note)*

Follow-Up Interview Guide

Introduction:
Hello, this is __________ calling for a follow-up interview as part of you participation in the study "Evaluating the Impact of an Intensive Educational Workshop on Evidence-Informed Decision Making (EIDM) Knowledge, Skills, and Behaviours."

We expect that this interview will take a maximum of 30 minute to complete. I will be asking you questions about your preferences for continuing education for knowledge and skills you gained during the week-long evidence-informed decision making workshop. The information obtained from this interview will be kept confidential and anonymous. Your comments will be combined with those of other respondents in such a way that they will not be able to be traced back to you. You have the right to terminate the interview at any point or to refuse to answer specific questions. Shall we begin?

If YES continue. If NO, attempt to arrange a convenient callback; for example: Is this a good time for you? May I call you back at another time? When would be a convenient time to call you back?

1. Generally, what are you thoughts on participating in the week-long evidence-informed decision making workshop?
2. Would you have liked to have additional learning opportunities after the week-long workshop ended? How so?
3. How would you feel about additional learning opportunities offered online?
4. Some additional online learning opportunities might include individual tutorials, journal club, or webcasts. Would you use these opportunities?
5. Another additional learning opportunity would be to have one-on-one contact with a "mentor". Would you be interested in this opportunity? How often would you like to have contact with a "mentor?
6. Generally, what are reasons that may motivate you to participate in additional learning opportunities?
7. How often can you realistically participate in additional learning opportunities, i.e. once month, three times a year? How would this change depending on the learning opportunity?
8. Generally, how much time would be reasonable for you to participate in additional learning opportunities at one time? How would this change depending on the learning opportunity?
9. Are there any other suggestions that you can provide for possible additional learning opportunities?
